# Bimetallic Nanoparticles as Efficient Catalysts: Facile and Green Microwave Synthesis

**DOI:** 10.3390/ma9070550

**Published:** 2016-07-08

**Authors:** Magda Blosi, Simona Ortelli, Anna Luisa Costa, Michele Dondi, Alice Lolli, Sara Andreoli, Patricia Benito, Stefania Albonetti

**Affiliations:** 1 ISTEC-CNR, Institute of Science and Technology for Ceramics, National Research Council, Via Granarolo 64, Faenza 48018, Italy; magda.blosi@istec.cnr.it (M.B.); simona.ortelli@istec.cnr.it (S.O.); anna.costa@istec.cnr.it (A.L.C.); michele.dondi@istec.cnr.it (M.D.); 2 Department Industrial Chemistry “Toso Montanari”, Bologna University, Viale Risorgimento 4, Bologna 40136, Italy; alice.lolli4@unibo.it (A.L.); sara-189-@hotmail.it (S.A.); patricia.benito3@unibo.it (P.B.)

**Keywords:** Au/Cu, Pd/Au, Pd/Cu, nanosols, microwave-assisted synthesis, supported catalysts, HMF, hydrodechlorination

## Abstract

This work deals with the development of a green and versatile synthesis of stable mono- and bi-metallic colloids by means of microwave heating and exploiting ecofriendly reagents: water as the solvent, glucose as a mild and non-toxic reducer and polyvinylpirrolidone (PVP) as the chelating agent. Particle size-control, total reaction yield and long-term stability of colloids were achieved with this method of preparation. All of the materials were tested as effective catalysts in the reduction of p-nitrophenol in the presence of NaBH_4_ as the probe reaction. A synergistic positive effect of the bimetallic phase was assessed for Au/Cu and Pd/Au alloy nanoparticles, the latter showing the highest catalytic performance. Moreover, monoand bi-metallic colloids were used to prepare TiO_2_- and CeO_2_-supported catalysts for the liquid phase oxidation of 5-hydroxymethylfufural (HMF) to 2,5-furandicarboxylic acid (FDCA). The use of Au/Cu and Au/Pd bimetallic catalysts led to an increase in FDCA selectivity. Finally, preformed Pd/Cu nanoparticles were incorporated into the structure of MCM-41-silica. The resulting Pd/Cu MCM-41 catalysts were tested in the hydrodechlorination of CF_3_OCFClCF_2_Cl to CF_3_OCF=CF_2_. The effect of Cu on the hydrogenating properties of Pd was demonstrated.

## 1. Introduction

In recent years, noble metal nanoparticles have attracted much attention owing to their potential application in many different fields due to their optical, electrical, mechanical and catalytic properties. Bimetallic nanoparticles, composed of two different metal elements, are of a great interest from both the scientific and technological points of view. The structure of bimetallic nanoparticles is defined by the distribution modes of the two elements and can be oriented in random alloy, alloy with an intermetallic compound, cluster-in-cluster and core-shell structures, leading to enhanced applications, compared to monometallic nanoparticles [1,2,3,4].

In this context, bimetallic nanostructures show remarkably improved optical, catalytic and electronic properties with respect to monometallic counterparts [5]. In fact, it is expected that bimetallic nanoparticles could show not only the combination of the properties related to the presence of two individual metals, but also new properties due to a synergy between the two metals. The shape and size of mono- and bi-metallic nanoparticles are strictly dependent on the preparation methods and conditions and affect the physicochemical properties of the final nanomaterial. The combination of two metals results in much more possibilities in shape and structure due to the miscellaneous distribution of each metal within a particle and their various organization [6].

Pre-formed metal nanoparticles can be used in catalysis in a wide range of reactions. The deposition of these particles onto different supports is fundamental to guarantee particles’ stability, mechanical resistance and easier work-up procedure at the end of the process. Thus, a synthetic approach, which allows a homogeneous nanoparticle suspension, is of crucial importance for catalytic applications, in order to exploit the real effect of the active phase on the activity. This effect is even more significant when it comes from the comparison of bimetallic and monometallic nanoparticles. In particular, the development of a colloidal synthesis that can guarantee precise composition, morphology and particle size is of crucial relevance for bimetallic nanoparticles [7].

In this study, we report the development of bimetallic nanostructures (Au/Cu, Pd/Au, Pd/Cu) by means of a patented environment-friendly approach [8], easily transferable to large-scale production and allowing the achievement of stable nanosols, even at high solid loading. Water is the benign solvent; glucose serves as a mild, renewable and non-toxic reducing agent; and polyvinylpyrrolidone (PVP) as a water-soluble, inexpensive and non-toxic chelating additive. The uses of inexpensive and nontoxic chemicals, environmentally-benign solvents and renewable materials are some of the essential issues in the nanomaterials science field in light of the “green” synthetic strategy for industrial-scale manufacturing [9]. Most of the synthetic routes reported in the literature, even if scalable to large production, are based on organic solvents, thus implying a complex environmental path to the industrial production [10,11,12,13,14]. So far, some expensive and/or toxic chelating agents (thiols, oleic acid, hexadecylamine, trioctylphosphine oxide) [15,16,17] are employed to prepare metal nanoparticles in organic solvents, making these synthesis processes less promising for a subsequent industrial scale up. Moreover, concerning the reducing agents, a large body of literature [18,19,20] proposed strong and hazardous reducing agents, such as hydrazine, sodium borohydride (NaBH_4_) and dimethyl formamide (DMF), which are highly reactive and present potential environmental and biological risks.

From the process intensification viewpoint, microwave irradiation was exploited as the heating source [21,22,23], so enabling rapid achievement of the desired temperatures and the homogenous heating of the treated volumes.

On all of the prepared bimetallic nanosols, the catalytic performances were assessed and compared exploiting the probe reaction of 4-nitrophenole (4-NP) hydrogenation [24]. Then, on the supported catalysts, a deeper catalytic characterization was carried out.

Gold-supported nanoparticles have been widely used as catalysts in the field of alcohol oxidation reactions [25,26]. More recently, these catalysts have been tested in 5-hydroxymethylfurfural (HMF) oxidation to produce 2,5-furandicarboxyic acid (FDCA) [27,28], a monomer used for the synthesis of a new class of polymers made from renewables [29,30]. In fact, HMF is derived from sugars’ dehydration, and it can be transformed into a series of interesting building blocks, both exploited in the pharmaceutical and in the chemical industries [31]. In order to improve catalyst performance in terms of activity and stability, researchers have focused their attention on the development of bimetallic nanoparticles with controlled size, composition and morphology and the use of different supports [7]. For example, Au/Pd-supported catalysts are considered to be efficient for alcohols oxidation reactions [32].

The goal of this paper was thus to describe the synthetic approach developed in our group. This microwave (MW)-assisted method allows the formation of colloidal monometallic and bimetallic nanoparticles with controlled size, composition and different morphologies (alloy and core-shell) to be used as is and for the preparation of supported catalysts. In particular, supported Au/Cu and Au/Pd nanoparticles were used in HMF oxidation, while Pd/Cu-MCM-41 catalysts were tested in the hydrodechlorination of CF_3_OCFClCF_2_Cl to produce trifluoromethyl trifluorovinyl ether (CF_3_OCF=CF_2_).

## 2. Results and Discussion

### 2.1. Preparation and Characterization of Metallic Sols

All of the prepared bimetallic sols exploited the green glucose as the reducing agent, which maximizes its reducing performance in alkaline conditions [33,34]. A simple, microwave-assisted strategy was developed for the preparation of different metal nanoparticles stabilized by polyvinylpyrrolidone (PVP), with various morphologies (alloy and core-shell). For lack of space, in this work, we will just discuss the results obtained using metallic alloys; nevertheless, recent works report our results obtained utilizing core-shell systems [35,36]. As a general observation, microwave heating has been shown to provide homogeneous particle nucleation and shorter synthesis time than traditional heating, and the developed synthesis route showed several advantages with respect to other methods (Figure S1). Indeed, it is simple, eco-friendly, carried out at low temperature and easily transferable to large-scale production [8]. Particle size-control, total reaction yield and long-term stability of colloids were achieved thanks to an accurate reaction optimization and the combination with microwave heating, promoting the intensification of the process even for large-scale production.

The characteristics of the synthesized bimetallic samples are listed in Table 1.

#### 2.1.1. Au/Cu System

Au and Cu nanosols showed very different colloidal stability. In fact, the Au sample showed an optimal time stability of almost 12 months, while Cu nanosol was affected by strong aggregation phenomena that involved precipitation within 24 h.

In detail, Au nanoparticles had a mean hydrodynamic diameter by Dynamic Light Scattering (DLS) of 17 nm, with a low polydispersity index (PDI) value typical of a narrow size distribution. On the contrary, Cu nanoparticles showed a bimodal distribution with the major peak set at 350 nm. This marked difference was not reflected by TEM analysis, which is reliable for the detection of Cu primary nanoparticles with an average dimension of 3 nm, so neglecting the coarser and sedimented particles.

The co-syntheses performed for achieving the alloy compounds, Au/Cu, showed a very good colloidal stability (several months), despite the aggregation tendency observed for Cu nanoparticles alone. Probably, the presence of Au seed acted with a sort of stabilizing effect, preventing the rapid aggregation of the newly-formed bimetallic particles. Anyway, the influence of the copper presence was observed by DLS. For gold and gold/copper suspensions, up to a Au:Cu atomic ratio 1:1, a relatively monodispersed signal, with an average diameter of 17–20 nm, was detected. Increasing the copper content led to polydispersed particles with a larger mean DLS diameter. Moreover, as indicated by the PDI, the size distribution was closely related to the average particle diameter: the larger the particle diameter, the broader the particle size distribution. Actually, these results seems to indicate that aqueous sols, up to a Au:Cu atomic ratio 1:1, are predominantly constituted by homogeneous bimetallic nanoparticles with a unimodal particle size distribution, while increasing the copper content in the sol causes the formation of nanoclusters with inhomogeneous size and/or composition, as shown by XRD analysis discussed in the following. Furthermore, a deepened study on the influence of PVP concentration over the hydrodynamic diameter was performed and reported in the Supplementary Materials (Table S1 and Figure S2).

XRD analyses with different Au:Cu molar ratios showed the peak broadening typical of nanosized crystallites. The patterns are consistent with the single-phase face centered cubic structure (fcc) of gold and copper (space group Fm-3m), with no observable impurities of CuO*_x_* of the phase-segregated metals up to the Au_1_Cu_3_ sample. Increasing the copper amount, a shift of the main gold peak was progressively observed; this behavior is consistent with the formation of the alloy compounds [39]. For higher Cu content (Au_1_Cu_3_, Au_1_Cu_6_), the presence of a reflection at 43.1° 2θ suggests the segregation of copper with the formation of copper oxide (Cu_2_O).

The interplanar distances assessed for the different samples are reported in Table 2. Probably in the presence of an excess of copper, only part of the Cu forms the alloy, while the remaining part could be oxidized by gold, forming a redox couple Cu/Au. The decreasing of the interplanar distance for a high copper amount would be caused by Cu-Au isomorphous substitution with copper atoms characterized by a lower atomic radius.

**Figure 1:**
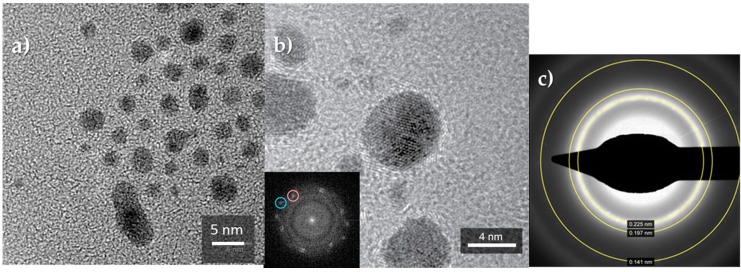
HRTEM images of sample Au_1_Cu_1_ at different magnifications (**a**,**b**). The inset shows the FFT of the observed nanoparticle (distances at 0.22 and 0.20 nm are circled); (**c**) selected area electron diffraction analysis (SAED) analysis performed on several nanoparticles.

Moreover, the bimetallic materials give broader XRD peaks, suggesting that the Au/Cu system has a smaller crystallite size than the monometallic one. In effect, crystal size values, estimated using the Scherrer equation, indicated ~4 nm for gold and ~3–3.5 nm for Au/Cu sols, confirming the coarser size of the monometallic system. These results were also confirmed by TEM analysis that evidenced for Au_1_Cu_1_ a particle size lower than the monometal Au, supporting the formation of the alloy Au_1_Cu_1_ (Figure 1). As well as the gold nanoparticles [40], also the bimetallic structure (Au_1_Cu_1_) showed a spherical morphology. The irregular shape observed for the larger particles probably results from the aggregation during the permanence for 40 min at 90 °C.

The selected area electron diffraction analysis (SAED) supported the formation of the Au/Cu alloy, corresponding to crystallites with a face centered cubic structure. The collected signals evidenced interplanar distances of 0.22, 0.20 and 0.14 nm, corresponding respectively to the plans (111), (200) and (220) and consistent both with the XRD results and with the values reported in the literature for the Au_1_Cu_1_ alloy (JCPDS Card No. 65-0937) (Table 3).

**Figure 2:**
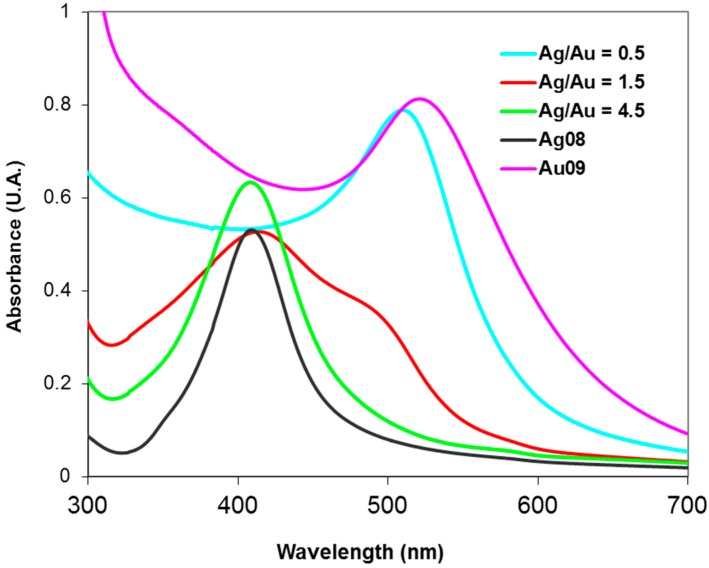
UV-VIS spectra collected for Au/Cu alloy samples.

The evolution of the surface plasmon resonance bands (SPR) for the Au/Cu samples is shown in Figure 2. By adding Cu, the SPR maximum peak absorption shifted from the Au characteristic value (525 nm) towards the Cu typical value, becoming even in shape more similar to the Cu resonance band characterized by a non-sharp profile [40].

The reaction yield for these samples was calculated by estimation of the unreacted ions assessed by ICP analysis on the ultrafiltered samples. The results showed a total reaction yield for all of the considered samples.

#### 2.1.2. Pd/Au and Pd/Cu Systems

Exploiting the same green reaction, both Pd/Au and Pd/Cu compounds were synthesized in the form of alloys. As observed for Au/Cu sols, despite the aggregation tendency of the only Cu nanoparticles, the coupling with Pd produced highly stable sols, evidencing a positive synergistic effect on the colloidal stability.

**Figure 3:**
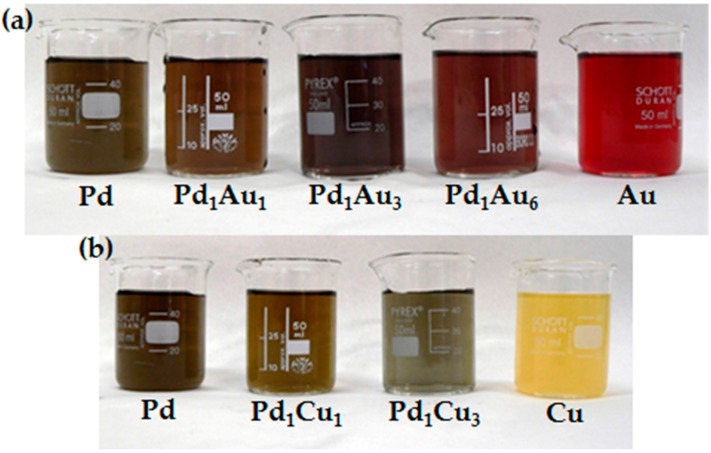
Images of (**a**) the Pd/Au series; and (**b**) the Pd/Cu series.

Figure 3 shows some of the prepared sols for both Pd/Au and Pd/Cu series. The addition of palladium, due to its typical dark color, with a very broad absorbance in the visible range, enhanced the darkness of the suspensions, making very difficult the collection of UV-VIS spectra for these nanosols.

The hydrodynamic diameters evaluated by DLS are listed in Table 1. For the Pd/Au sols, the resulting curves were monomodal with mean diameters comparable each other (from 24 to 35 nm) and slightly larger than the single metal ones (17 nm for Au and 23 nm for Pd). Only Pd_1_Au_2_ evidenced a larger dimeter, 71 nm, probably due to some aggregation phenomena.

**Figure 4:**
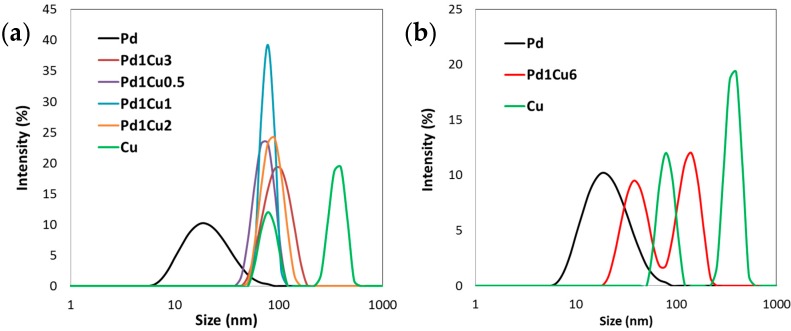
Particle size distribution assessed for Pd/Cu bimetallic nanosols: (**a**) samples from Pd_1_Cu_0.5_ to Pd_1_Cu_3_ compared with Pd and Cu; (**b**) Pd_1_Cu_6_ sample compared to Pd and Cu.

A different behavior was observed for the Pd/Cu series, as already shown in Au/Cu samples; the higher is the Cu amount, the larger is the detected hydrodynamic diameter (from 73 to 133 nm) with an increased polydispersion well evidenced by the PDI values. The size distribution curves (Figure 4a,b) showed the marked influence produced by the copper addition, which for the composition Pd_1_Cu_6_ evidenced the bimodal size distribution typical of copper sols.

**Figure 5:**
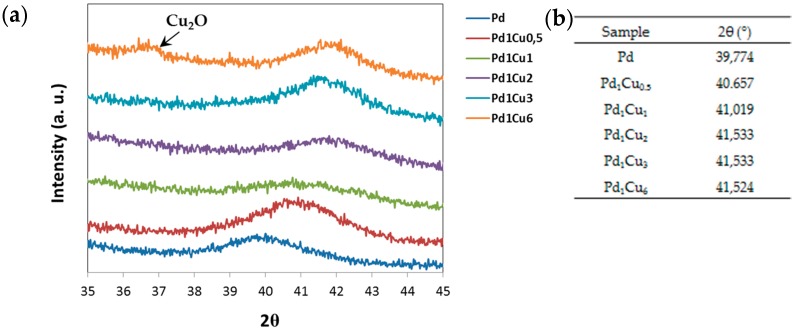
(**a**) Focusing of the XRD spectra on the principal peak for the Pd/Cu series; (**b**) List of the progressive shift of the principal detected peaks. Progressive shift of the principal detected peak.

We hypothesized that the smaller peak could corresponded to a different phase, for example Cu_2_O; probably, with a large excess of copper the total reduction of the precursor is too difficult to be completed, and an oxidized part remained in the sample. This hypothesis was strengthened by the XRD results that evidenced the formation of Cu_2_O in the sample containing the highest amount of Cu (Figure 5).

In Figure 5, both the diffraction pattern collected for the Pd/Cu samples (a) and the table listing the shifts detected for the principal peak that supports the formation of the alloys (b) are reported.

Anyhow, the peak shift was well appreciated till the Pd_1_Cu_2_ sample, but for a copper molar concentration higher than 67%, the data suggested that the added copper does not enter the lattice structure, but contributes to the segregation phenomenon of Cu_2_O. On sample Pd_1_Cu_6_, the analysis of the data confirmed the presence of two different phases; in fact, the main peak at 36.5° 2θ corresponds to the Cu_2_O oxide phase (JCPDS Card No. 65-3288), while at 41.524° 2θ, the detected peak corresponds to the PdCu alloy (JCPDS Card No. 48-1551).

**Figure 6:**
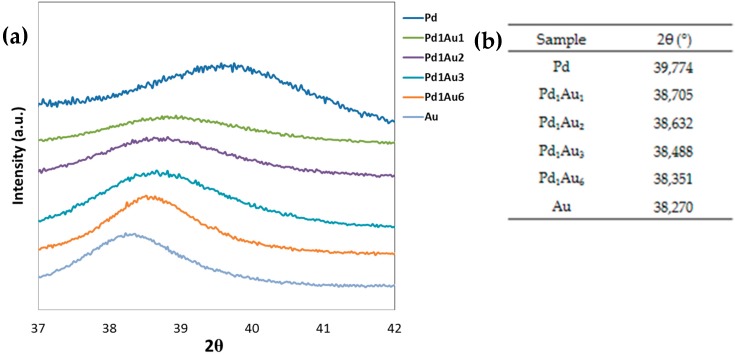
(**a**) Focusing of the XRD spectra on the principal peak for the Pd/Au series; (**b**) List of the progressive shift of the principal detected peaks.

By means of the Scherrer equation, the mean crystallite size was calculated and reported in Table 1. Except for Pd_1_Cu_0.5_, the results are coherent with the trend observed by DLS data; indeed, the higher was the copper amount, the larger was the mean crystallite size. The peak shift was detected also for the Pd/Au series, and for this sample, no segregated phases were detected; the progressive shift is consistent with the formation of PdAu alloy (Figure 6).

### 2.2. Catalytic Activity

#### 2.2.1. Catalytic Test of Pristine Nanoparticles in the Hydrogenation of 4-Nitrophenol

**Scheme 1:**
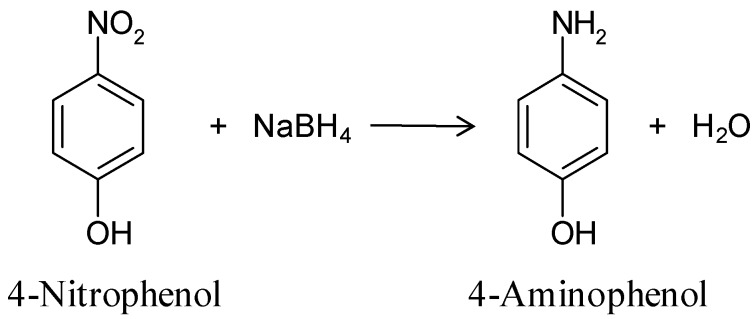
4-Nitrophenol reduction with NaBH_4_.

The catalytic performance of the synthesized nanoparticles was tested in the reduction of 4-nitrophenol (4-NP) to 4-aminophenol (4-AP) with an excess of NaBH_4_, as the model reaction (Scheme 1). Accordingly, the reduction rates can be regarded as being independent of the concentration of the reducing agent, NaBH_4_ [41].

This reaction is particularly easy to follow because only the product 4-aminophenol (4-AP) is formed, and the extent of the reaction can be determined by measuring the change in UV-VIS absorbance at the 400-nm wavelength typical of 4-NP in alkaline condition.

Preliminary tests without the catalyst indicated that no reaction occurs in the studied conditions, confirming the absence, during our tests, of non-catalytic phenomena. On the contrary, all of the nanoparticles acted as effective catalyst for the hydrogenation of 4-nitrophenol in the presence of NaBH_4_. Indeed, the addition of metallic sols to the reaction mixture causes a gradual decoloration of the solution and ultimately a bleaching of the yellow color, due to the formation of 4-AP, showing a weak absorption of violet wavelength stemming from its peak at 300 nm.

**Figure 7:**
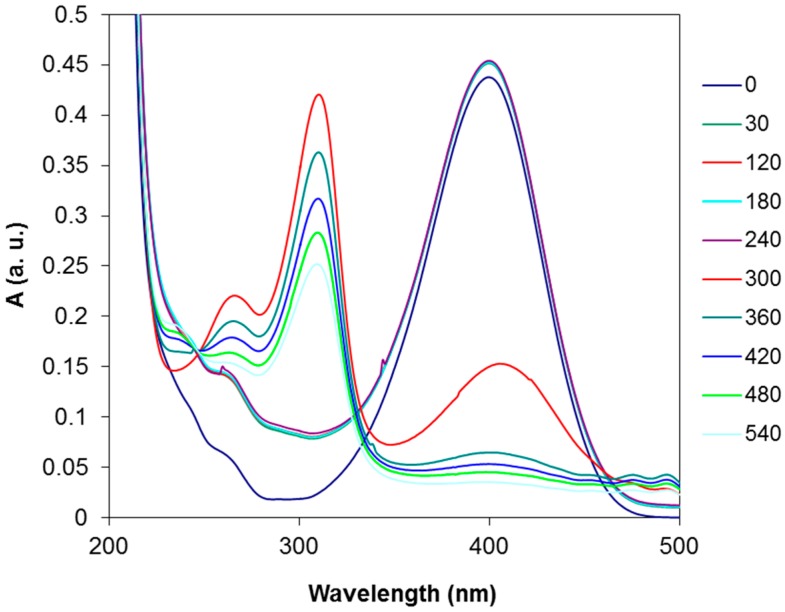
Typical evolution of UV-VIS spectra as a function of time during the hydrogenation of 4-nitrophenole (4-NP) in the presence of catalyst.

In Figure 7, typical degradation spectra of 4-nitrophenolate are reported. On the basis of the collected UV-VIS spectra, the plot of ln (A/Ao) versus time was obtained and the kinetic constants calculated. The resulting kinetic constants for all of the bimetallic nanosols are listed in Table 4.

As reported elsewhere [42], the reaction does not start immediately after the addition of the catalyst, but only after a noticeable time lag, i.e., the induction time. This may be ascribed to several reasons: the presence of oxygen dissolved in water or a thin nanoxide coating on the particle surface. In order to reduce the induction time, several strategies have been investigated [40,43,44], observing that it was significantly reduced for all of the prepared samples only by bubbling nitrogen for 1 min before the addition of NaBH_4_ and adding the reagents in this order: catalyst, 4-NP and, finally, NaBH_4_. Probably, this procedure allows the removal of oxygen dissolved in the solution, which would involve NaBH_4_ in a competitive reaction with 4-NP. The results, referring to data collected after the pre-treatment with bubbling nitrogen, are summarized in Table 4 in terms of kinetic constant, conversion and turn over frequency (TOF, defined as the number of moles of reduced 4-NP per mole of catalyst per second).

**Figure 8:**
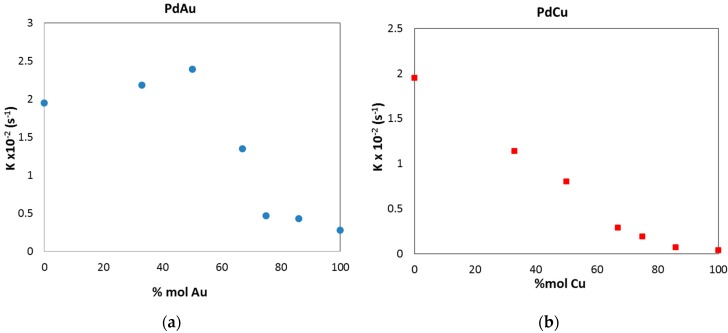
Evolution of the kinetic constant for the 4-NP hydrogenation reaction for Pd bimetallic compounds with different amounts of Au and Cu added: (**a**) the Pd/Au system and (**b**) the Pd/Cu system. Data refer to the following concentration: 4-NP 9 × 10^−5^ M, catalyst 1.1 × 10^−6^ M, NaBH_4_ 0.715 M.

All of the prepared nanoparticles act as catalysts in the hydrogenation of 4-NP; among the single metals Au, Cu and Pd, the best performances were observed for Pd, followed by Au and Cu. As evidenced in the literature [45,46], bimetallic nanoparticles could show not only the combination of the properties related to the presence of two individual metals, but also new properties due to the so-called synergistic effect between the two metals. A marked synergistic effect on the catalytic performances for this probe reaction was evidenced for Au/Cu and Pd/Au nanostructures. In Au/Cu, despite the poor catalytic performance assessed for the Cu monometal, the addition of Cu to Au produced an enhancement of the activity for the composition of Au_1_Cu_1_. A further increase of Cu content produced, instead, a detrimental effect. The latter was justified by the marked increment of the hydrodynamic diameter and the formation of Cu_2_O segregated phases. Similarly, in Pd/Au series, the synergic effect was assessed till the composition Pd_1_Au_1_, which reached the maximum performance (Figure 8a). Again, a further increase of added Au metal decreases progressively the catalytic activity, despite the similar nanoparticle dimension, hydrodynamic diameter and homogeneity of the bimetallic phase (Table 1).

On the other hand, the Pd/Cu compounds did not show any kind of catalytic improvement with respect to the single Pd metal. Indeed, a constant decreasing trend was observed adding Cu (Figure 8b) combined with structural variation, such as the hydrodynamic diameter increase and the occurrence of the segregation phenomena (Table 1), which probably prevailed on any potential synergistic outcome.

It is well know that the synergistic effect on the catalytic activity depends on the surface electronic structure that can be modified by the interactions between the two kinds of atoms in the bimetallic alloy owing to the strain and ligand effects. The “strain effect” involves the average bond length of metal atoms, which is typically different from those in the monometals, while the so-called “ligand effect” involves the hetero-metallic bonding interactions of atoms in the surface of nanoparticles, which modifies their surface electronic structure [6]. Even if the mechanism is still under debate, both effects result in changes in the width and average energy of the d-band that may account for the enhancement in catalytic activity.

The hydrogenation reaction involving 4-NP can be divided into two main parts: firstly, the decomposition of NaBH_4_ on the surface of nanoparticles, producing H-atoms, which turn to be available to 4-NP molecules, and, finally, the addition of protons to the 4-NP and the simultaneous removal of its oxygen, following one of the typical mechanisms of heterogeneous reactions, like the Eley–Rideal or the Langmuir Hinshelwood [42]. An overall comparison among the catalysts can be carried out on the basis of the TOF value, which evidenced for the Pd-based catalysts the best catalytic performances, probably due to the Pd intrinsic characteristic of efficient hydrogen relay.

Instead, comparing the catalytic performances for the Pd-based catalysts, the most active compound resulted in being Pd_1_Au_1_. As hypothesized for Au/Pt alloys [46], also for the Au/Pd nanostructure, a synergic effect between neighboring Pd and Au sites could be triggered. Indeed, it was proposed that, during the hydrogenation of nitroaromatic compounds, the Pd clusters adsorb BH_4_-/H_2_ dissociatively with a much lower activation barrier than Au. Thus, activated H atoms can migrate to Au or mixed Pd/Au sites located in the neighborhood. The same co-operative effect could act also between Au and Cu, supporting the synergistic behavior shown for the Au_1_Cu_1_ alloy [47]. Furthermore, some structural features described in the literature for the Au/Cu bimetallic nanoparticles [48] ascribe to the composition Au_1_Cu_1_ the formation of the most ordered and packed structure at the nanoscale, with the smallest crystallite size.

#### 2.2.2. Catalytic Tests of Supported Catalysts in the Oxidation of 5-Hydroxymethylfurfural

**Scheme 2:**
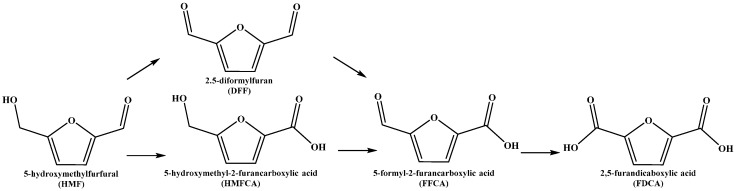
General reaction scheme for 5-hydroxymethylfufural (HMF) oxidation.

5-hydroxymethyfurfural (HMF) can be used to prepare different intermediates, and 2,5-furandicarboxylic acid (FDCA) is considered to be one of the most interesting derivatives from the chemical point of view. In Scheme 2, a general reaction scheme for HMF oxidation is reported. Generally, using gold-based nanoparticles, the first transformation that occurs is the oxidation of the aldehydic group, generating the first intermediate 5-hydroxymethyl-2-furancarboxylic acid (HMFCA). Most of the time, the rate-limiting step for this process is the oxidation of the alcoholic group; however, when 5-formyl-2-furancarboxylic acid (FFCA) is formed, it suddenly reacts to form the desired product 2,5-furandicaboxylic acid (FDCA) [28,35,49,50]. 2,5-diformylfuran (DFF) is not observed in the most commonly-used oxidation conditions, since it is converted very fast into the second intermediate FFCA.

Preformed mono- and bi-metallic nanoparticles were supported onto TiO_2_ and CeO_2_ and used as heterogeneous catalysts for 5-hydroxymethylfufural (HMF) oxidation to 2,5-furandicarboxylic acid (FDCA) in water. The synthetic approach reported in the previous section allowed the preparation of reproducible and stable colloids with a controlled particle size, and the influence of the metal active phase on the catalytic activity was studied, since the catalyst preparation procedure did not affect the catalyst performance.

**Figure 9:**
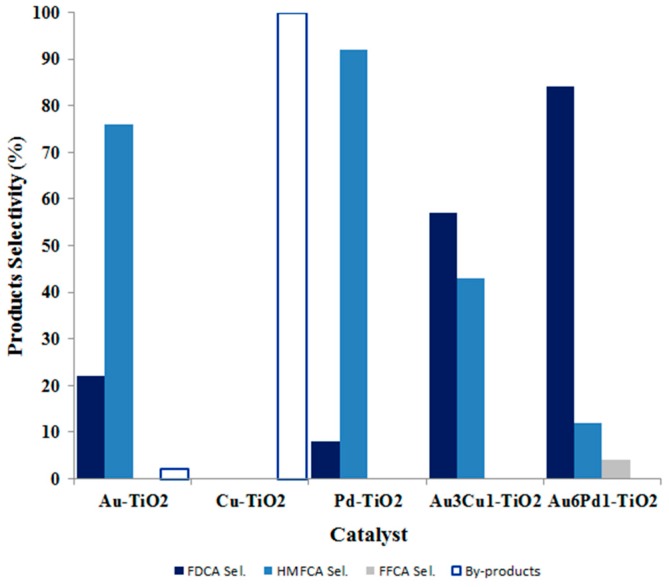
Product selectivity achieved using different metal active phases under mild reaction conditions (70 °C, 10 bar O_2_, H_2_O as solvent, NaOH, HMF:metal 1:0.01, 4 h); results are given at total conversion of HMF. Data report catalysts’ performance in their best condition (Au- and Cu-containing samples have been tested using four equivalents of NaOH, while Pd-containing samples required only two equivalents of the base).

Monometallic Au-, Cu- and Pd-supported catalysts were used in HMF oxidation, and their catalytic activity was compared to that typical of bimetallic systems (Au/Cu, Au/Pd). The obtained results confirm that catalyst activity and product selectivity is affected by the presence of bimetallic nanoparticles as the active phase. Indeed, our recent works demonstrated that the ability of synthesizing bimetallic systems with a different metal molar ratio can be the key to tune catalyst selectivity towards a specific compound. Moreover, the presence of an alloy can improve catalyst activity. The HMF oxidation reaction can be exactly the case where the presence of an alloy improves greatly the catalytic performance. As a matter of fact, Au/Cu- and Au/Pd-based catalysts are even three-times more active than the monometallic counterparts. The graph reported in Figure 9 underlines the effect of alloyed nanoparticles on the catalytic activity. As far as monometallic catalysts are concerned, both Au and Pd are active in HMF oxidation, while Cu-supported material did not allow substrate transformation, leading to the formation of by-products derived from HMF degradation. However, the addition of very low amounts of copper and palladium in the system is able to increase FDCA production under mild reaction conditions. It can be noticed that a specific metal molar ratio must be employed to achieve the highest FDCA yield: the Au/Cu sample characterized by a metal molar ratio 3:1 gave actually the best performance. The selectivity towards the desired product (FDCA) increased significantly for Au_3_Cu_1_ and Au_1_Cu_1_, which emphasizes the importance of the Au:Cu ratio to achieve copper promotion on gold. Our data suggest that Cu acts as a gold promoter and/or dispersing agent only if present in a Au:Cu ratio higher than 1:1, and the highest FDCA yield can be obtained using a ratio of Au:Cu of 3:1 [38].

**Figure 10:**
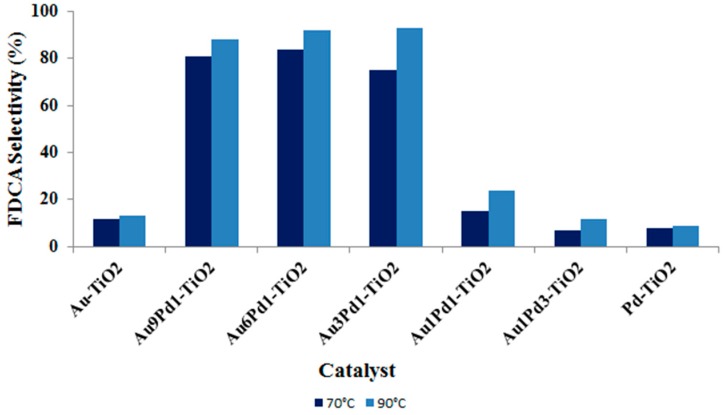
FDCA selectivity for TiO_2_-supported Au/Pd catalysts at different reaction temperatures. Reaction conditions: 10 bar O_2_, H_2_O as solvent, HMF:NaOH:metal 1:2:0.01, 4 h. Results are given at total conversion of HMF.

**Figure 11:**
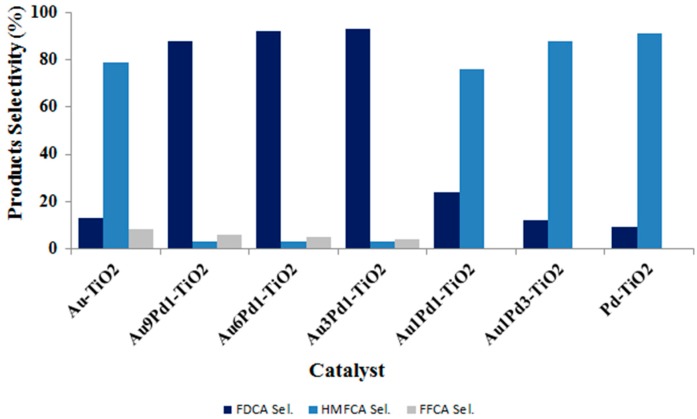
Products selectivity for TiO_2_-supported Au/Pd-based catalysts. Reaction conditions: 90 °C, 10 bar O_2_, H_2_O as solvent, HMF:NaOH:metal 1:2:0.01, 4 h. Results are given at total conversion of HMF.

Products’ distribution was also strongly affected by the Au:Pd atomic ratio (Figure 10 and Figure 11). In particular, the results clearly showed the presence of a synergic effect due to the presence of the two metals with increased FDCA selectivity due to the increase in Au content, until the Au:Pd atomic ratio was equal to 1:6 (Figure 10). The further increase of Au content resulted in lower FDCA selectivity, although all catalysts with high Au content (Au_3_Pd_1_, Au_6_Pd_1_ and Au_9_Pd_1_) were more efficient in the formation of this product [35]. These results confirm the recent data reported by Prati and co-workers who used Pd/Au nanoparticles supported on activated carbon [50] for glycerol and alcohol oxidation. Therefore, a small quantity of Pd seems to be adequate to significantly change the electronic properties of Au and enhance the catalytic activity in alcohol oxidation.

The temperature increase from 70 to 90 °C scarcely affected FDCA selectivity (Figure 10). Indeed, for the reported Au/Pd systems, the tuning of the metal molar ratio seems to be significantly more important than the increase in reaction temperature. Furthermore, even the product distribution was slightly affected by temperature. Indeed, the 20 degree temperature increase led only to a slight increase of FDCA formation with a simultaneously decrease of the amount of HMFCA in the final reaction mixture (Figure 11). The oxidation of the primary alcohol side-chain, necessary to form the FDCA, is a more demanding process and proceeds with a significant rate at temperatures higher than 80 °C. Therefore, this step is favored by the use of Au/Pd-supported catalyst with high gold content, confirming once again the importance of synthesizing the bimetallic active phase with a specific metal molar ratio.

**Figure 12:**
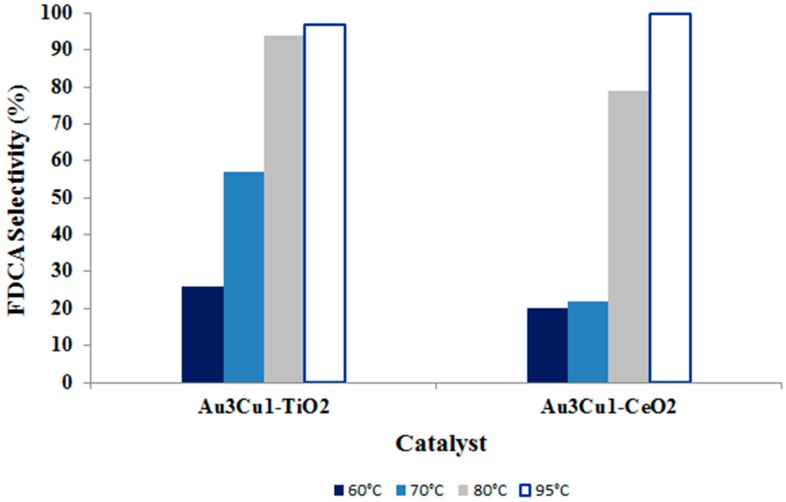
FDCA selectivity achieved using the Au/Cu catalyst in the ratio of 3:1 supported on TiO_2_ and CeO_2_ at different temperatures. Reaction conditions: 10 bar O_2_, H_2_O as solvent, HMF:NaOH:metal 1:4:0.01, 4 h. Results are given at total conversion of HMF.

Although the type of metal site played a pivotal role, it must be noticed that even the support can strongly influence the catalytic activity in HMF oxidation. As an example, the use of cerium oxide to support bimetallic nanoparticles had a detrimental effect on FDCA production with respect to TiO_2_ samples (Figure 12). Indeed, in the case of cerium oxide, the presence of a second metal in the active phase seems to prevent the interaction between gold and ceria support, which was demonstrated to be the key factor for HMF oxidation over this system [51]. However, these differences can be overcome increasing the reaction temperature: at 95 °C, the FDCA yield is around 100% for both systems.

**Figure 13:**
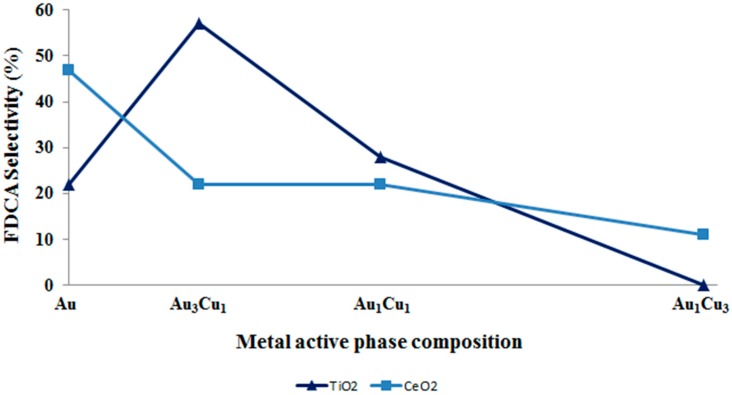
FDCA selectivity as a function of copper content in bimetallic catalyst when TiO_2_ and CeO_2_ are used as the support. Reaction conditions: 70 °C, 10 bar O_2_, H_2_O as solvent, HMF:NaOH:metal 1:4:0.01, 4 h. Results are given at total conversion of HMF.

These data highlighted that the rate-limiting step of the reaction (alcohol oxidation) can be promoted either by the use of the appropriate metal phase or by the increase in temperature. The right active phase that has to be used depends on the chosen support. Generally, using titania to support bimetallic nanoparticles, an enhancement in the catalytic activity can be achieved especially when metals are alloyed in the ratio Au:Cu 3:1. On the other hand, it seems not to be worth using bimetallic nanoparticles when ceria is used as the support. Au/Ce is the best catalyst, and the addition of copper in different amounts worsens the activity (Figure 13). Using cerium oxide-supported catalysts, a proper interaction between gold and the support must be guaranteed to strength the catalytic activity. This means that, in order to design a catalyst with improved FDCA production, the support effect must be taken into consideration and the choice of the best active phase depends on the selected support.

**Figure 14:**
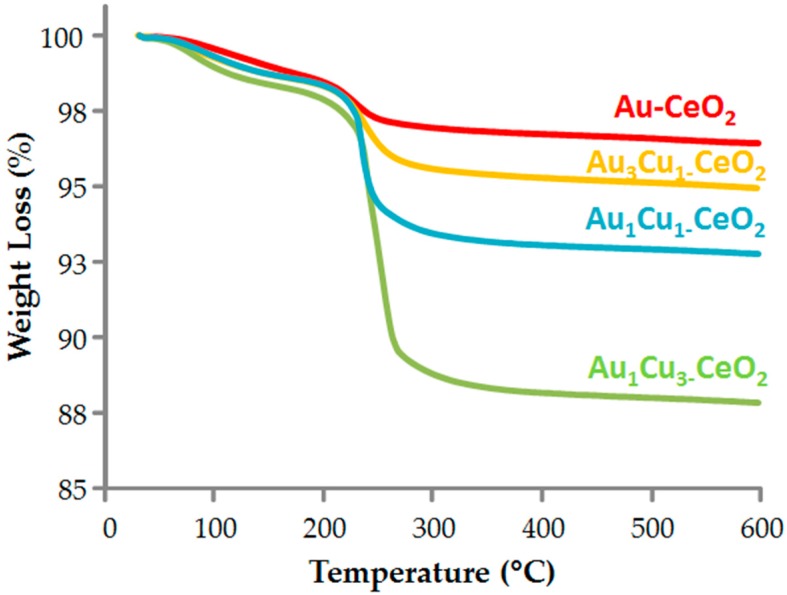
TGA curves for Au/Cu-CeO_2_ catalysts.

With the aim of increasing FDCA production, the prepared catalysts have been calcined at different temperatures. Our recent data [51] demonstrated that both with Au-Ce and for Au_3_Cu_1_-Ce samples, catalytic activity can be increased by removing polyvinylpyrrolidone (PVP) from the catalyst surface. In fact, metal nanoparticles, deposited onto the support, are surrounded by polyvinylpyrrolidone, the stabilizing agent used during the synthetic procedure (as reported in Table 5 in the Materials and Method section), which can actually prevent a proper interaction between the substrate and the metal active site and the deep contact between the metal and the support. TGA curves reported in Figure 14 show an increase in catalyst weight loss due to PVP combustion with the copper content. This is due to the presence of a higher amount of PVP on Au/Cu samples.

**Figure 15:**
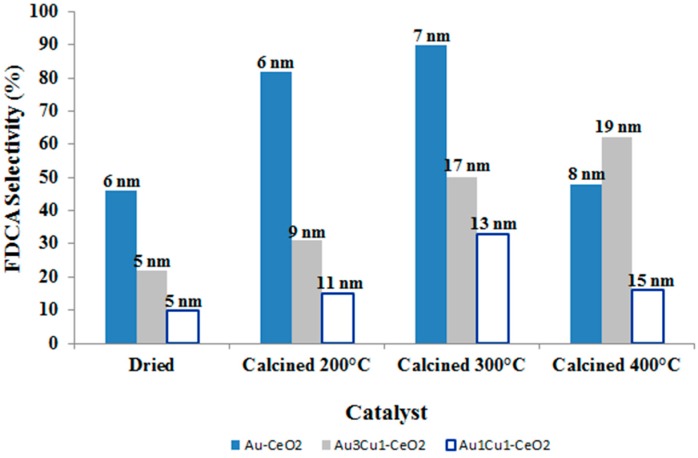
FDCA selectivity and particle size distribution (from XRD analysis) as a function of calcination temperature. Reaction conditions: 70 °C, 10 bar O_2_, H_2_O as solvent, HMF:NaOH:metal 1:4:0.01, 4 h. Results are given at total conversion of HMF.

Due to the enhanced interaction of metal nanoparticles with reactants and support, once PVP was removed by combustion, Au-Ce and AuCu-Ce catalysts were calcined in static air at different temperatures, and their catalytic results are reported in Figure 15. Calcination at 300 °C led to the increase of the FDCA yield for almost every sample, because this temperature allows the complete PVP combustion, as shown in TGA analysis. The beneficial effect of the improved interaction of the substrate with the metal is particularly evident for the Au-Ce catalyst. Moreover, this catalyst is characterized by the presence of a smaller amount of residual PVP from the gold synthesis, and consequently, it is less affected by particle growth. As a matter of fact, particle sintering is fostered by the local exothermicity generated during PVP combustion, and it is directly correlated to PVP content. The calcination of the catalyst at 200 °C did not promote HMF oxidation, since this temperature is not high enough to remove the stabilizing agent. On the contrary, the increase in the calcination temperature up to 400 °C worsened the catalytic performance, basically because of particles’ growth. Moreover, in bimetallic systems, some segregation effects must be taken into account, which can modify the catalytic properties. However, from the data reported in Figure 15, it is evident that even bigger particles are active in HMF oxidation; this could be due to the presence of OH^−^ in water solution that promotes alcohol oxidation even with 20-nm particles [52]. In fact, the hydroxyl absorption on the Au surface is considered to be fundamental to increase the activity of Au-supported catalysts [53,54,55].

Thus, the catalytic activity is influenced by PVP removal (which brings an enhanced interaction active phase-support), particle growth, phase segregation (which actually modifies the metal active phase) and OH^−^ concentration in aqueous phase.

**Figure 16:**
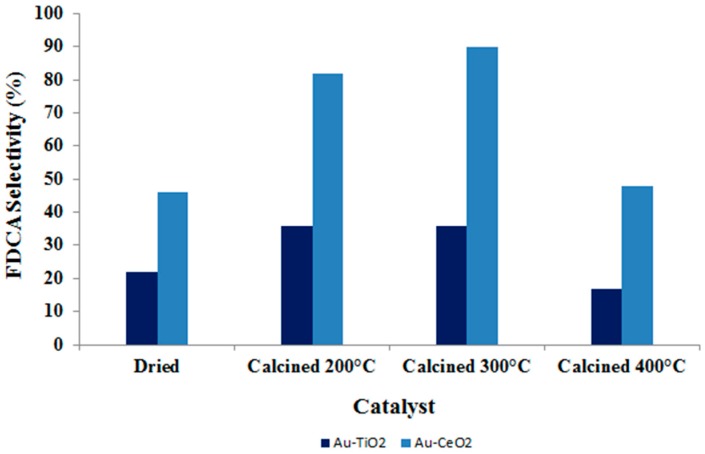
FDCA selectivity as a function of calcination temperature. Reaction conditions: 70 °C, 10 bar O_2_, H_2_O as solvent, HMF:NaOH:metal 1:4:0.01, 4 h. Results are given at total conversion of HMF.

In Figure 16, the comparison of the catalytic activity between Au-Ce and Au-Ti calcined catalysts is reported. Even if FDCA selectivity increases for the catalyst calcined at 300 °C, the improvement due to thermal treatment is not as evident as it is for Au-Ce, highlighting once again the differences between the support used.

#### 2.2.3. Catalytic Tests in the Hydrodechlorination of CF_3_OCFClCF_2_Cl

Today, the commercial production of trifluoromethyl trifluorovinyl ether (CF_3_OCF=CF_2_) is based on CF_3_OCFClCF_2_Cl precursor dechlorination [56] and produces large quantities of by-products, such as ZnCl_2_. A new approach, based on H_2_-assisted gas-phase dechlorination over metal-supported catalysts, may lead to a new sustainable process [57,58].

Hydrogen-assisted dechlorination (elimination of halogen atoms to form unsaturated compounds) and hydrodechlorination (replacement of selected halogen atoms with hydrogen) have received much attention in the literature as an option for the safe disposal of chlorofluorocarbon and to produce valuable chemicals. Monometallic Pd, Ru or Pt materials were reported to be effective for the hydrodechlorination of dichloroethane to ethylene, but this useful product was readily converted into ethane over these catalysts [59]. Conversely, group IB-based monometallic Sn or Cu catalysts are effective for the full dechlorination of chlorinated hydrocarbons with higher selectivity toward ethylene; however, such catalysts are less active under mild conditions and can be easily deactivated [60]. For this reaction, bimetallic catalysts, such as Pt/Cu, Pt/Sn, Pd/Cu and Pd/Ru [61], have been proven to be much more selective toward ethylene than the monometallic noble metals.

Based on this information, in our recent work, we investigated the production of trifluoromethyl trifluorovinyl ether by the hydrogen-assisted dechlorination of CF_3_OCFClCF_2_Cl using bimetallic Pd/Cu catalysts supported over mesostructured silica. Since the hydrodechlorinating performances were demonstrated to be strongly related to the Pd/Cu interaction and the size of metallic particles, Pd/Cu-MCM-41 catalysts were prepared from pre-formed Pd/Cu nanoparticles, to verify the possibility of reaction optimization [62].

**Scheme 3:**
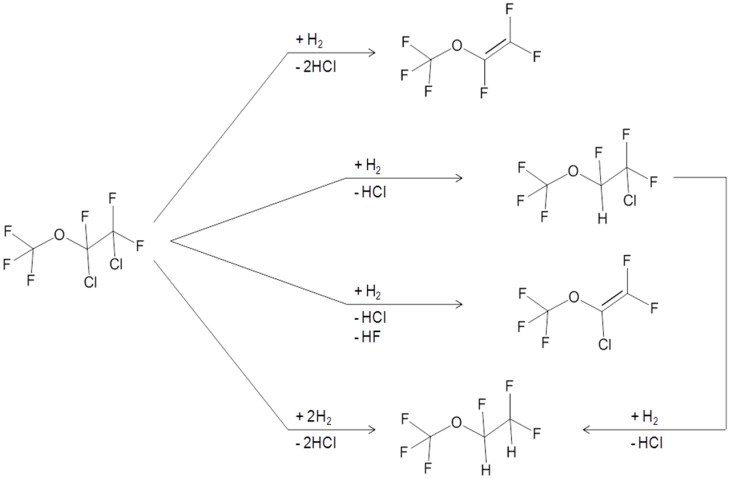
General reaction scheme for CF_3_OCFClCF_2_Cl hydrodechlorination.

Scheme 3 reports the main products detected during the catalytic tests. The main product, CF_3_OCF=CF_2_ (MVE), Cl/H substitution by-products like CF_3_OCFHCF_2_Cl and CF_3_OCFClCF_2_H (AMH), de-fluorinated by-products like CF_3_OCCl=CF_2_ (AMH*) and products such as CF_3_OCFHCF_2_H (AMH_2_) were formed in different quantities depending on the catalytic systems used.

PdCu-MCM-41 catalysts were demonstrated to be able to activate H_2_ and to anchor the chlorofluorinated reagents. Moreover, the presence of Cu decreases the Pd hydrogenating activity, increasing the selectivity in the desired product [62,63]. However, catalytic performances were demonstrated to depend on the synthesis procedure, silica source and template removal procedure. Indeed, catalyst preparation determined the size and composition of metallic particles and Pd/Cu interaction.

**Figure 17:**
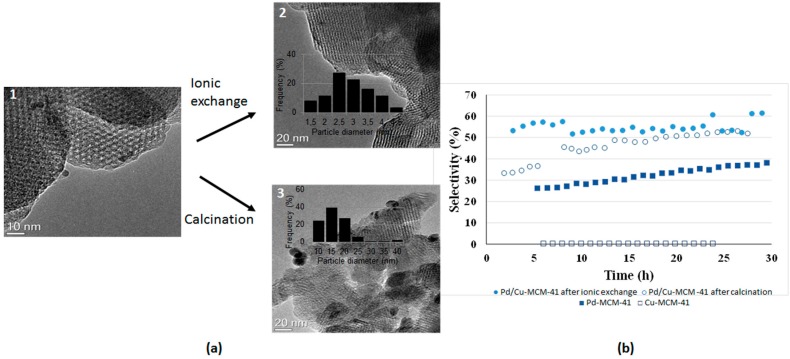
(**a**) TEM images of the Pd/Cu-MCM-41 sample before the template removal (1), after the ionic exchange (2) and the calcination (3); (**b**) selectivity in the desired product (CF_3_OCF=CF_2_) as a function of time.

The hexagonal structure of MCM-41 (Figure 17a), the large specific surface area (1035 m^2^·g^−1^) and the pore volume (1.275 cm^3^·g^−1^) were preserved after the inclusion of active species using Pd/Cu preformed nanoparticles and led to small and well-dispersed bimetallic species in the MCM-41 structure. However, template removal by calcination caused both the increase of the particle size and the oxidation of the metal. As a consequence, most of the investigated materials contained different species: (i) Pd/Cu mixed species; (ii) well-dispersed copper oxide; and (iii) segregated Pd species.

Pd/Cu species were demonstrated to be mainly responsible for the formation of the desired product (CF_3_OCF=CF_2_), while the segregated Pd and Cu species caused different selectivity in side reactions (Figure 17b). Indeed, a high amount of palladium led to the formation of hydrogenated molecules, such as CF_3_OCFHCF_2_H, while particles enriched in palladium promoted the formation of CF_3_OCH=CF_2_. In addition, Pd/Cu nanoparticles with a low content of Pd induce F/Cl substitution with the production of a significant amount of CF_3_OCCl=CF_2_.

To avoid the segregation of the oxides during template removal, ionic exchange [64] was used rather than calcination, demonstrating the possibility to control the properties of bimetallic Pd/Cu-MCM-41 through the careful choice of the template elimination treatment.

## 3. Materials and Methods

The following analytical grade reagents were used for sols and supported catalysts preparation: HAuCl_4_·3H_2_O, CuSO_4_·5H_2_O, NaOH, AgNO_3_, PdCl_2_, polyvinylpyrrolidone PVP (Mwa 29,000), all from Sigma-Aldrich (Sigma-Aldrich, St. Louis, MO, USA), and D(+)glucose (Merck, Darmstadt, Germany), TiO_2_ anatase (DT51 Millennium Chemicals, Baltimore, MD, USA), CeO_2_ (Evonik VP AdNano 90, Essen, Germany). Metallic nanoparticles were prepared following a patented procedure [8], which provides stable nanosols by using a microwave-assisted eco-friendly method. MCM-41-supported catalyst were prepared by using sodium silicate as the silica source and cetyltrimethylammonium bromide (99%, Sigma-Aldrich, CTAB) as the structure-directing template. The synthetic procedures involved the preparation of the gel, hydrothermal treatment and calcination, as reported in a previous paper [65]. The following reagents were used for catalytic reactions: 4-nitrophenol (Sigma Aldrich), NaBH_4_ (Sigma Aldrich), 5-hydroxymethylfurfural (Alfa Aesar, Karlsruhe, Germany).

### 3.1. Nanoparticles Synthesis

Metal nanoparticles were prepared using microwave heating. The microwave system used was a MicroSYNTHplus (Milestone, Bergamo, Italy), whose reaction chamber is provided with magnetic stirring, a reflux system and an optical fiber temperature controller. The microwave power is generated by 2× 800-W magnetrons with a frequency of 2.45 GHz. In order to follow the scheduled heating ramp, the power is continuously supplied and automatically modulated by software; for each ramp, only the maximum deliverable power can be imposed.

- Au colloids: In a typical reaction, PVP (0.35 g) and glucose (0.40 g) were mixed in a round-bottomed flask containing water (80 mL), and the solution was heated at ambient pressure using microwave stimulation to 90 °C (heating rate 30 °C·min^−1^). At this temperature, aqueous NaOH (10 mL, 0.88 M) and HAuCl_4_ (10 mL, 0.11 M) were added to the flask and stirred for 2.5 min. After the reaction, a red, stable suspension of gold nanoparticles with a concentration of 0.011 M (0.2 wt %) was obtained.
- Cu colloids were obtained through the reduction of CuSO_4_·5H_2_O. In a typical experiment, PVP (5.66 g), glucose (7.2 g) and NaOH (1.2 or 2.4 g) were mixed in a round-bottomed flask containing water (180 mL), and then, the solution was microwave heated at ambient pressure to 100 °C (heating rate 30 °C·min^−1^). At this temperature, the CuSO_4_·5H_2_O solution (20 mL, 0.165 M) was added, and the temperature was maintained for a time ranging from 10–40 min. In order to achieve the pure metallic phase, both the NaOH/metal molar ratio and reaction time were set as reported in Table 5. A red sol with a solid loading of 0.1 wt % and a time stability of 24 h was obtained.
- Au/Cu colloids: The bimetallic colloids, Au/Cu, were obtained through the microwave (MW)-assisted co-reduction of HAuCl_4_ and CuSO_4_·5H_2_O. Au/Cu were prepared on the basis of the experimental set up used for the monometal particles, in order to work with the conditions the most useful for both metals. For this reason PVP, glucose and NaOH contents were optimized for each sample depending on the Au/Cu ratio and with the purpose to ensure the total formation of the copper metallic phase, the reaction time was set to 40 min. However, in order to decrease the nanoparticle size, some syntheses were performed with a reaction time of 2.5 min. In a typical reaction for preparing Au_1_Cu_1_ sol, PVP (1 g), NaOH (0.52 g) and glucose (1.27 g) were mixed in a round-bottomed flask containing water (180 mL), and the solution was heated at ambient pressure to 90 °C (heating velocity 30 °C·min^−1^) using microwaves. At this temperature, a solution containing both HAuCl_4_ (20 mL, 0.025 M) and CuSO_4_ (0.025 M) with a Au/Cu molar ratio of 1 was added to the flask with stirring, and the temperature was maintained for 40 or 2.5 min. The samples were prepared with Au/Cu molar ratios of 3/1, 1/1, 1/3, 1/6 (Table 5) and a total solid concentration of 0.005 M. After the reaction, a brown, stable suspension of bimetallic nanoparticles was obtained.
- Pd colloids were synthesized through the reduction of PdCl_2_. The dissolution of PdCl_2_ was facilitated by adding 3 drops of concentrated HCl (37%) and by keeping the solution at 90 °C till complete dissolution. The experiments were performed following the same procedure used for gold colloids, but with a different metal concentration.
- Pd/Cu and Pd/Au colloids: In a typical preparation of the Pd/Cu system, 0.062 g of CuSO_4_·5H_2_O were dissolved in 5 mL of water and 0.044 g of PdCl_2_ in 10 mL of H_2_O. The so-obtained precursor solutions were mixed together and then added under stirring to 85 mL of the microwave heated (90 °C) solution containing 0.51 g of PVP, 0.64 g of glucose and 0.26 g of NaOH. The reaction was performed for 2.5 min, and the synthesized samples were produced by keeping the total metal concentration at 0.005 M and respecting the molar ratio listed in Table 5. The same procedure was followed for the Pd/Au colloids.

### 3.2. Supported Catalysts Preparation

Monometallic and bimetallic catalysts supported on TiO_2_ and CeO_2_ were prepared by immobilization on the support surface of the preformed colloids. Before use, the as-prepared sols were concentrated and washed with distilled water using 50-KDa Amicon Ultra filters (Millipore) to eliminate the excess PVP and other reagents dissolved in the aqueous media, and then, the colloids were impregnated onto the supports with the incipient wetness impregnation method. For all samples, the solvent was evaporated by thermal treatment at 120 °C. The total metal loading of the catalyst was always 1.5 wt %. The catalyst samples are denoted as Au-Ti, Cu-Ti, Pd-Ti and Au*_x_*Cu*_y_*-Ti, Pd*_x_*Au*_y_*-Ti, Au*_x_*Cu*_y_*-Ce, where x and y refer to the Au:Cu or Pd:Au molar ratio and Ti and Ce refer to the TiO_2_ and CeO_2_ supports (i.e., Au_1_Cu_1_-Ti indicates a TiO_2_-supported sample synthesized with a Au:Cu molar ratio of 1).

Pre-formed Pd/Cu colloids were included in the MCM-41 structure in the course of the synthesis of the mesoporous material by a microwave-assisted synthesis. In particular, CTAB was solubilized in the nanoparticle solution prepared by the reduction of CuSO_4_ and PdCl_2_ with D(+)glucose as reported above; then, sodium silicate was added [62]. Slurries were microwave-hydrothermally treated at 125 °C for 7 h. The template was removed by calcination at 540 °C or with ionic exchange using an ethanolic solution of NH_4_NO_3_.

### 3.3. Characterization of Metallic Sols

Metallic nanosols were characterized by optical spectroscopy (UV-VIS), dynamic light scattering (DLS), X-ray diffraction (XRD), inductively-coupled plasma-atomic emission spectrometry (ICP-AES) and transmission electron microscopy (TEM). UV-VIS extinction spectra were measured with a Lambda 35 spectrophotometer (Perkin Elmer, Waltham, MA, USA), using a quartz cuvette as the sample-holder. Samples for UV-VIS spectroscopy were prepared by diluting the as-prepared colloidal suspension with water in order to get into the cuvette the same metal concentration for every sample.

Dynamic light scattering (DLS) was used to monitor the hydrodynamic diameter and the particle size distribution of the suspensions. Measurements were carried out by Nano S (Malvern, Malvern, UK) working at the fixed angle of 173°. Samples were properly diluted with water and poured in a polystyrene cuvette before measurement. The hydrodynamic diameter includes the coordination sphere and the species adsorbed on the particle surface, such as stabilizers, surfactants and so forth. DLS analysis provides also a polydispersion index parameter (PDI), ranging from 0 to 1, quantifying the colloidal dispersion degree; for PDI below 0.2, a sol can be considered monodispersed.

Diffraction patterns were collected on the synthesized samples dripped on a glass slide and dried at 100 °C for 15 min. Analyses were performed by the Bruker D8 Advance diffractometer (Bruker, Karlsruhe, Germany) operating in θ/2θ configuration, with a LynxEye detector (20°–80° 2θ range, 0.02 step size, 16 s time-per-step equivalent). The quantitative phase composition was performed by using the Reference Intensity Ratio method RIR.

In order to calculate the reaction yield, the ionic fraction was separated from the sample and analyzed by chemical analysis (ICP). Fifteen milliliters of suspension were typically centrifuged using the ultra-centrifugal filter (UCF) unit (Amicon Ultra-15, 10 kDa, Millipore, Billerica, MA, USA), with a centrifugal force of 5000 rpm and a spin time of 30 min. The UCF unit allows retaining NPs and filtrating away the unreacted ions; this way, 10 mL of filtered solvent, which contains the dissolved metal ions, were subjected to elemental analysis using inductively coupled plasma-optical emission spectrometry (ICP-OES) (Liberty 200, Varian, Santa Clara, CA, USA).

The observation of the particle size distribution and morphology was performed using a transmission electron microscope (TEM, Tecnai F20, FEI, Hillsboro, OR, USA). Nanoparticles were dispersed on a standard copper grid for TEM by dripping the prepared solutions, which were dried in air then treated at 100 °C for 5 min.

### 3.4. Characterization of Supported Catalysts

To verify the behavior of different catalysts under thermogravimetric analysis, TGA was obtained using a Rheometric Scientific STA1500 analyzer while heating the sample in air from 25 °C–600 °C.

### 3.5. Catalytic Tests

- Probe reaction with 4-Nitrophenol: The catalytic reduction of 4-nitrophenol by NaBH_4_ was studied at room temperature (25 °C) in a standard quartz cuvette with a 1-cm path length and about a 3-mL volume. The prepared samples were properly diluted with distilled water in order to achieve a metal concentration of 1.1 × 10^−2^ mM. Thus, 10 mL of diluted suspensions were mixed with 5 mL of a 4-nitrophenol solution (9.0 × 10^−2^ mM) and with 1 mL of a NaBH4 aqueous solution (0.72 M). An aliquot of the solution was poured into the quartz cuvette, and the absorption spectra were collected by a Lambda 35 spectrophotometer (Perkin Elmer, Waltham, MA, USA) in the range between 250 and 500 nm. The rate constants of the reduction process were determined by measuring the change in absorbance at 400 nm, corresponding to the 4-nitrophenolate ion, as a function of time. Although the 4-NP solution absorbs at 317 nm, a second peak appears at 400 nm after the addition of the NaBH4 solution, forming the 4-nitrophenolate ion. The reaction is of second order (1), but as the concentration of NaBH4 is in large excess with respect to the reagent (4-NP) one, the reduction rate can be regarded as independent from NaBH4, changing the system in a pseudo first order reaction (2). Therefore, the rate constants of the reaction can be determined by measuring the change in absorbance at 400 nm, the wavelength typical of 4-NP in alkaline condition, as a function of time.
- 5-hydroxymehtyfurfural (HMF) oxidation: HMF oxidation was carried out in a 100-mL autoclave reactor (Parr Instrument) equipped with a mechanical stirrer (0–600 rpm) and tools for the measurement of temperature and pressure. The reactor was charged with 25 mL of distilled water, the appropriate amount of 5-hydroxymethylfufural, NaOH and catalyst (HMF/metal molar ratio = 100). The autoclave was purged 3 times with O_2_ (5 bar) and then pressurized at 10 bar. If not differently indicated, the temperature was set at 70 °C, and the reaction mixture was stirred at 400 rpm for 4 h. At the end of the reaction, the reactor was cooled down to room temperature, and the solution was filtered, diluted 5 times and analyzed with an Agilent Infinity 1200 liquid chromatograph equipped with a Aminex HPX 87-H 300 mm × 7.8 mm column using a 0.005 M H_2_SO_4_ solution as the mobile phase. The identification of compounds was achieved by calibration using reference commercial samples.
- Catalytic hydrodechlorination of CF_3_OCFClCF_2_Cl: The hydrogen-assisted dechlorination of CF_3_OCFClCF_2_Cl to CF_3_OCF=CF_2_ was performed in a 10 mm diameter down-flow Hastelloy^®^ tubular reactor. The catalysts were pelletized and reduced before catalytic tests. A mixture of CF_3_OCFClCF_2_Cl (19% *v*/*v*), H_2_ (19% *v*/*v*) and N_2_ was fed to the reactor for 20 h of time-on-stream with a contact time of 10 s at 250 °C and 1 atm. The obtained products were sampled before a water scrubber and analyzed with a TCD-equipped gas chromatograph.

## 4. Conclusions

A green and versatile approach was developed to produce stable mono- and bi-metallic colloids (Au/Cu, Pd/Au, Pd/Cu) with a total reaction yield. The method was optimized by means of microwave heating and exploiting ecofriendly reagents: water as the solvent, glucose as a mild and non-toxic reducer and PVP as the chelating agent. Compositional and particle size-control were achieved and widely supported by UV-VIS, DLS, TEM and XRD analyses; moreover, the so-prepared systems exhibited long-term stability.

All of the prepared particles acted as effective catalysts in the reduction of 4-NP to 4-AP, exploited as the probe reaction. The catalytic activity observed in the first probe test showed significant differences among the samples, evidencing for the Pd-based catalysts the best performances and noticeable synergistic effects for AuCu and PdAu alloys at intermediate composition.

Furthermore, the obtained mono- and bi-metallic colloids, employed to prepare TiO_2_- and CeO_2_-supported catalysts, were tested for the liquid phase oxidation of 5-hydroxymethylfufural (HMF) to 2,5-furandicarboxylic acid (FDCA). Au/Cu and Au/Pd bimetallic catalysts led to an increase in FDCA selectivity, demonstrated for specific metal molar ratios. Even the choice of support was important to enhance the catalytic properties of the metal active phase.

Finally, preformed Pd/Cu nanoparticles were incorporated into the structure of MCM-41 silica. The resulting Pd/Cu MCM-41 catalysts were tested in the hydrodechlorination of CF_3_OCFClCF_2_Cl to CF_3_OCF=CF_2_, evidencing that the presence of Cu decreases the Pd hydrogenating activity, so increasing the selectivity in the desired product.

The preparation of supported bimetallic catalysts using size-controlled preformed nanoparticles, whose particle size is established before the deposition and/or incorporation on the metal oxide support, was demonstrated to be a very efficient synthetic procedure. Nevertheless, the optimized temperature of the thermal treatment must be carefully chosen in order to preserve particle size and morphology, avoiding sintering and phase segregation effects.

## Figures and Tables

**Table 1:** Particle size distribution of the synthesized nanoparticles by means of different techniques: dynamic light scattering (DLS), transmission electron microscopy (TEM), X-ray diffraction (XRD).

Sample ^1^	*d*-DLS (nm)	PDI ^3^	*d-*TEM(nm)	*d*-XRD (nm)
Au	17	0.2	4 ± 2 [37]	4 [38]
Cu	350, 80 ^2^	0.4	3 ± 1	70
Pd	23	0.3	2 ± 1 [35]	6
Au_3_Cu_1_	17 [38]	0.2	-	4.0 [38]
Au_1_Cu_1_	23	0.3	3 ± 2	3.5 [38]
Au_1_Cu_3_	61	0.3	-	3.0 [38]
Au_1_Cu_6_	80	0.5	-	n.d. [38]
Pd_3_Au_1_	-	-	3 ± 1 [35]	3
Pd_1_Au_0.5_	36	-	-	5
Pd_1_Au_1_	25	0.4	3 ± 1 [35]	3
Pd_1_Au_2_	71	-	-	3
Pd_1_Au_3_	35	0.3	4 ± 2	4
Pd_1_Au_6_	34	0.2	3 ± 2 [35]	5
Pd_1_Au_9_	-	-	4 [35]	5
Pd_1_Cu_0.5_	73	0.3	-	5
Pd_1_Cu_1_	79	0.4	-	3
Pd_1_Cu_2_	88	0.4	-	4
Pd_1_Cu_3_	99	0.2	-	5
Pd_1_Cu_6_	133, 40 ^2^	0.5	-	6

^1^ The nominal molar distribution of the bimetallic structure is reported; ^2^ the DLS diameter refers to the bimodal distribution; ^3^ polydispersion index.

**Table 2:** Interplanar distances (100) detected for Au, Cu and Au/Cu compounds.

Sample	d (Å)
Au	2.352
Au_1_Cu_1_	2.314
Au_1_Cu_3_	2.291
Au_1_Cu_6_	2.278
Cu	2.080

**Table 3:** Interplanar distances of the Au_1_Cu_1_ sample. The values collected by TEM and XRD are compared to the tabulated data.

Interplanar Distance	d (Å) TEM	d (Å) XRD	d (Å) Literature
d _(111)_	2.27	2.31	2.23
d _(200)_	1.97	2.03	1.98
d _(220)_	1.41	1.42	1.40
d _(311)_	1.20	1.22	1.19

**Table 4:** Catalytic performances of the prepared bimetallic nanosols. TOF, turn over frequency.

Sample	Catalyst Concentration × 10^−5^ (M)	K × 10^−2^ (s^−1^)	TOF × 10^−2^ (s^−1^)
Au	1.1	1.70	5.3
Cu	1.1	0.20	0.4
Pd	0.11	1.95	34.1
Au_1_Cu_1_	1.1	2.70	5.8
Au_1_Cu_3_	1.1	1.70	4.9
Au_1_Cu_6_	1.1	0.70	1.2
Pd_1_Cu_0.5_	0.11	1.14	32.2
Pd_1_Cu_1_	0.11	0.80	15.7
Pd_1_Cu_2_	0.11	0.26	7.8
Pd_1_Cu_3_	0.11	0.19	5.5
Pd_1_Cu_6_	0.11	0.07	3.1
Pd_1_Au_0.5_	0.11	2.10	40.7
Pd_1_Au_1_	0.11	2.29	49.5
Pd_1_Au_2_	0.11	1.35	37.5
Pd_1_Au_3_	0.11	0.59	30.3
Pd_1_Au_6_	0.11	0.35	10.3

Data refer to the following concentrations: 4-NP 9 × 10^−5^ M and NaBH_4_ 0.715 M. For all of the samples, TOF values are calculated considering the first 60 s of the reaction.

**Table 5:** The composition and the concentration of prepared bimetallic nanoparticles and the molar ratio with reagents used during the synthesis.

**Sample**	**%Au (mol/mol)**	**%Cu (mol/mol)**	**Cu/Au (mol/mol)**	**PVP/[Metal] (mol/mol)**	**Glucose/[Metal] (mol/mol)**	**NaOH/[Metal] (mol/mol)**	**[Metal] (mol/L)**
Au	100	0	-	3	2	8	0.011
Au_3_Cu_1_	75	25	0.3	6	5	11	0.005
Au_1_Cu_1_	50	50	1	9	7	13	0.005
Au_1_Cu_3_	25	75	3	12	10	16	0.005
Au_1_Cu_6_	16	84	6	13	10	17	0.005
Cu	0	100	-	15	12	18	0.017
**Sample**	**%Pd (mol/mol)**	**%Au (mol/mol)**	**Pd/Au (mol/mol)**	**PVP/[Metal] (mol/mol)**	**Glucose/[Metal] (mol/mol)**	**NaOH/[Metal] (mol/mol)**	**[Metal] (mol/L)**
Pd	100	0	-	2.75	2.00	8.00	0.005
Pd_3_Au_1_	75	25	3	2.75	2.00	8.00	0.005
Pd_1_Au_0.5_	67	33	2	2.75	2.00	8.00	0.005
Pd_1_Au_1_	50	50	1	2.75	2.00	8.00	0.005
Pd_1_Au_2_	33	67	0.5	2.75	2.00	8.00	0.005
Pd_1_Au_3_	25	75	0.33	2.75	2.00	8.00	0.005
Pd_1_Au_6_	14	86	0.16	2.75	2.00	8.00	0.005
Pd_1_Au_9_	10	90	0.11	2.75	2.00	8.00	0.005
**Sample**	**%Pd (mol/mol)**	**%Au (mol/mol)**	**Pd/Au (mol/mol)**	**PVP/[Metal] (mol/mol)**	**Glucose/[Metal] (mol/mol)**	**NaOH/[Metal] (mol/mol)**	**[Metal] (mol/L)**
Pd_1_Cu_0.5_	100	0	2	6.83	2.00	11.33	0.005
Pd_1_Cu_1_	67	33	1	8.88	5.37	13.00	0.005
Pd_1_Cu_2_	50	50	0.5	10.92	7.05	14.67	0.005
Pd_1_Cu_3_	33	67	0.33	11.94	8.74	15.50	0.005
Pd_1_Cu_6_	25	75	0.16	13.25	9.58	16.57	0.005

## Supplementary Materials

The following are available online at www.mdpi.com/1996-1944/9/7/550/s1.
