# Supplementary material for: Bimetallic Nanoparticles as Efficient Catalysts: Facile and Green Microwave Synthesis
Source: Materials (Basel). 2016 Jul 8;9(7):550. doi: 10.3390/ma9070550 (PMC5456855; doi:10.3390/ma9070550)
Supplement: Supplementary file 1 [file materials-09-00550-s001.pdf]

Supplementary Materials: Bimetallic Nanoparticles as Efficient Catalysts: Facile and Green Microwave Synthesis

Magda Blosi, Simona Ortelli, Anna Luisa Costa, Michele Dondi, Alice Lolli, Sara Andreoli, Patricia Benito and Stefania Albonetti

1. Microwave Heating Role

The microwave system provides important advantages in terms of heating homogeneity, in fact by dielectric stimulation the whole volume reaches the same temperature in few minutes so avoiding temperature gradients within the liquid volume. This situation is very useful to improve the control over nucleation and growth phenomena so promoting the contemporary nucleation of metal nanoparticles and preventing the excessive growth and aggregation of prepared nanoparticles. As a result, very often the same product prepared by traditional heating showed larger and broader particle size distribution, with a shorter stability over time. In Figure S1 the size distribution assessed by DLS for Ag nanosol, prepared at a concentration of 0.5 wt % with the microwave-assisted glucose method, was compared with the size distribution of the same material achieved by traditional heating systems (flask and mantle and beaker and heating plate). Passing from microwave to traditional heating the average hydrodynamic diameter ranges from 40 to 150 nm for the flask/mantle, till 250 nm for the beaker/heating plate setup. Similarly, the time stability was dramatically decreased from 24 months to 2 months till 48 h.

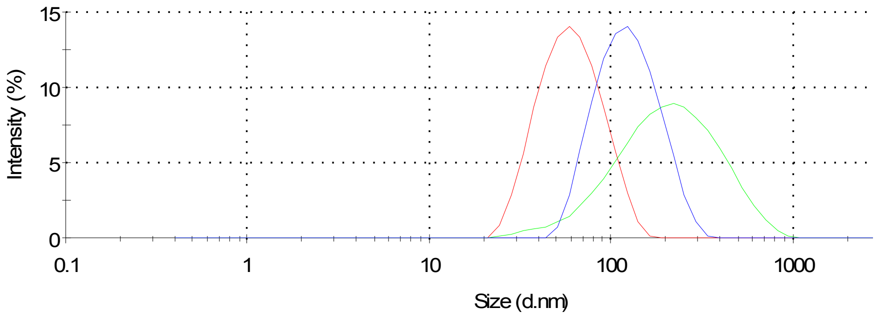

Figure S1. DLS particle size distribution assessed for Ag colloids (0.5 wt %) prepared by different heating methods: microwave (red line), flask and heating mantle (blue line), beaker and heating plate (green line).

2. PVP Influence on the DLS Measurements for Au/Cu

It was known that PVP adsorbed on the surface of nanoparticles affects the average hydrodynamic diameter, as well as the particle size distribution of the prepared nanosols. Since Au/Cu catalysts were prepared with different PVP amounts depending on the Au/Cu alloy composition, on these samples a deeper study was performed in order to investigate the influence of the different PVP concentration. At this purpose DLS analyses of the water solutions containing only PVP at the corresponding concentration were carried out and here reported.

Table S1. Hydrodynamic average diameters assessed for PVP water solutions corresponding to the concentrations employed in Au/Cu catalysts.

Sample	*d*-DLS (nm)	PDI
PVP (Au_1Cu_1)	18	0.4
PVP (Au_1Cu_3)	17	0.3
PVP (Au_1Cu_6)	17	0.3

The measurements evidenced no significant variations both in the average hydrodynamic dimeter (Table S1) and in the particle size distribution (Figure S2a) passing from the PVP concentration used in Au_1Cu_1 samples till Au_1Cu_6 (Table S1). In fact the average diameters were detected for all the considered samples at 17 or 18 nm with an intermediate polydispersion index of 0.3–0.4. Figure S2b–d compared the size distribution between the only PVP-water solution with the corresponding nanosol. As expected, the size distributions changed in the presence of the nanoparticles, depending on the dimensions of the synthesized solid phase. Anyway, passing from only polymer to polymer-particles, the main peak was maintained as shape and as dimension range. Probably in the presence of the solid particles the polymer, here used as chelating agent, changes its arrangement adsorbing on the surface of the particles. Basically, the resulting hydrodynamic diameter represents the diameter of the nanoparticle including the coordination sphere and the species adsorbed on its surface.

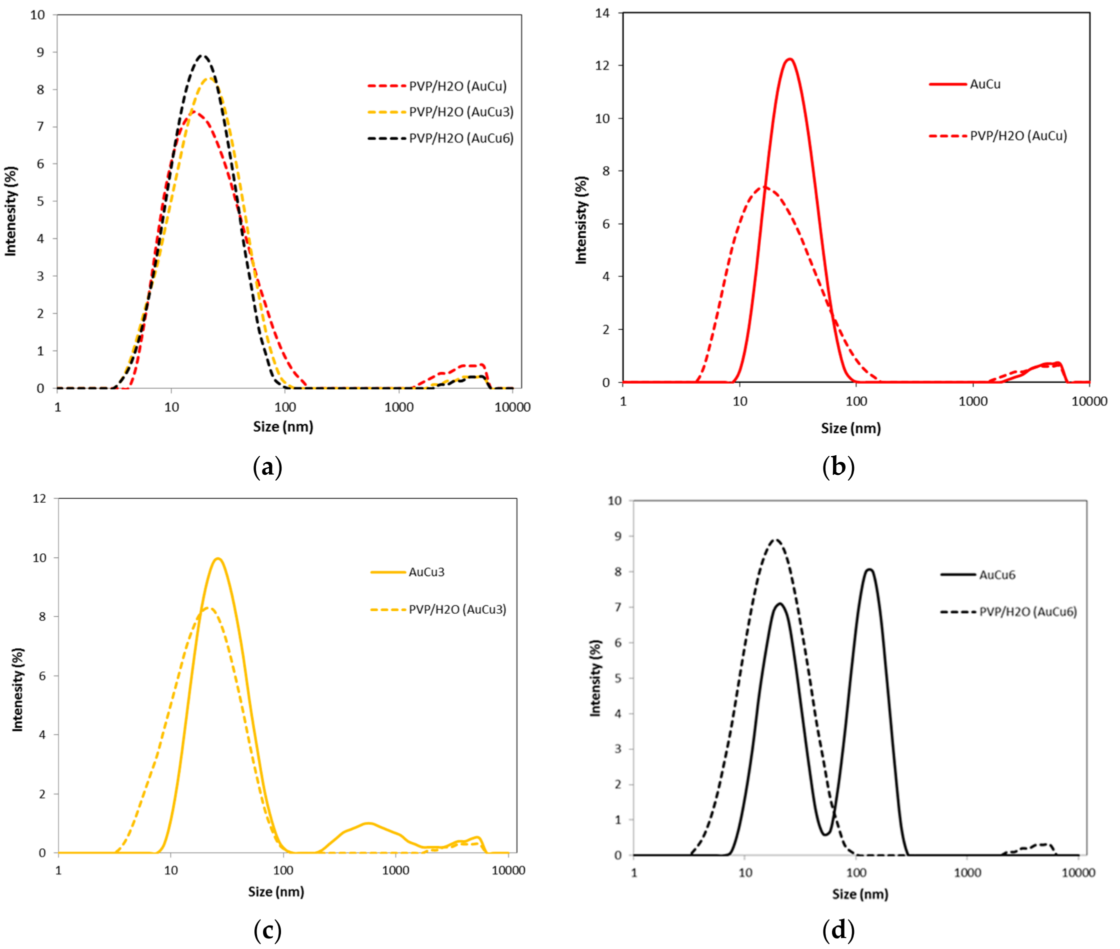

Figure S2. (**a**) Particle size distribution of PVP water solutions at different concentration of PVP, comparison of the size distribution of: (**b**) PVP in water with the corresponding Au_1Cu_1 nanosols; (**c**) PVP in water with the corresponding Au_1Cu_3 nanosols; (**d**) PVP in water with the corresponding Au_1Cu_6 nanosols.
